# Effects of Photodynamic, Nanoparticle-Based, and Chemical Final Post-Space Conditioning on Smear Layer Removal and Push-Out Bond Strength of Glass Fiber-Reinforced Composite Posts

**DOI:** 10.3390/polym18212762

**Published:** 2026-11-06

**Authors:** Mohammad H. Alrefeai, Fahad Alkhudhairy

**Affiliations:** Restorative Dental Sciences Department, College of Dentistry, King Saud University, Riyadh 11421, Saudi Arabia; malrefeai@ksu.edu.sa

**Keywords:** smear layer removal, push-out bond strength, superparamagnetic iron oxide nanoparticles, antimicrobial photodynamic therapy, apple cider vinegar

## Abstract

To evaluate and compare the effect of different final post space treatments, i.e., methylene blue-mediated antimicrobial photodynamic therapy (aPDT-MB), superparamagnetic iron oxide nanoparticles (SPIONs), and apple cider vinegar (ACV), against the established benchmark of 17% EDTA on smear layer (SL) removal and push-out bond strength (PBS) of glass fiber posts (GFPs) bonded to post space dentin. Endodontic treatment was conducted on eighty human mandibular premolars with single roots, which were obturated. Post spaces were uniformly prepared, and specimens were randomly divided into four groups based on the final post space treatment protocol (*n* = 20 per group): Group 1 (NaOCl + 17% EDTA), Group 2 (NaOCl + aPDT-MB), Group 3 (NaOCl+ SPIONs), and Group 4 (NaOCl + ACV). All teeth received conventional root canal treatment, including a final 17% EDTA rinse, before post-space preparation. Within each group, ten teeth were allocated to SL evaluation by scanning electron microscopy (SEM) and ten to PBS testing, in which GFPs were luted with a dual-cure self-adhesive resin cement. The two outcomes were therefore measured in different teeth, and antimicrobial efficacy was not assessed. The teeth underwent thermocycling (10,000 cycles, 5–60 °C) and were sectioned into cervical, middle, and apical segments. These segments were subjected to PBS testing at a speed of 0.5 mm/min using a universal testing machine (*n* = 10), and failure modes were determined through stereomicroscopic analysis. PBS data were analyzed using a two-way mixed ANOVA followed by simple-effects analyses with Tukey- and Bonferroni-adjusted comparisons; ordinal SL scores were analyzed using Kruskal–Wallis (between protocols) and Friedman (between root thirds) tests with Bonferroni-adjusted pairwise comparisons (α = 0.05). Group 3 (NaOCl + SPIONs) showed the lowest SL scores (medians 1.20–1.32) and the highest PBS values (10.70 ± 0.76 to 11.20 ± 0.84 MPa; maximum at the cervical third). Group 2 (NaOCl + aPDT-MB) showed the highest SL scores (medians 2.60–3.54) and the lowest bond strength (3.90 ± 0.52 to 6.10 ± 0.58 MPa). PBS ranged from 7.20 to 10.90 MPa in Group 1 and from 6.10 to 7.90 MPa in Group 4. No statistically significant difference was detected between NaOCl + EDTA and NaOCl + SPIONs at the cervical and middle thirds, whereas at the apical third NaOCl + SPIONs showed lower SL scores and higher PBS (mean difference 3.50 MPa; 95% CI 2.75–4.25). Combined NaOCl and SPIONs irrigation did not differ significantly from NaOCl + EDTA in the cervical and middle thirds and outperformed it in the apical third; this promising approach requires further validation, including assessment of residual nanoparticles, biological safety, and long-term bond durability.

## 1. Introduction

The restoration of endodontically treated teeth frequently requires intraradicular post placement to provide coronal support and mechanical retention for the definitive prosthetic reconstruction [1]. Among available systems, glass fiber posts (GFPs) have gained widespread clinical acceptance because their elastic modulus closely approximates that of root dentin, which may reduce the risk of catastrophic root fracture associated with rigid metallic counterparts and promote more favorable stress distribution along the post–dentin interface [2]. Fracture behavior, however, also depends on the remaining tooth structure, ferrule, post geometry, cementation, and loading conditions. Their long-term success, however, depends critically on the quality of the adhesive bond at the post–cement–dentin interface, which is in turn governed by the physicochemical condition of the post-space dentin walls at the time of cementation [3].

From a materials science perspective, glass fiber posts (GFPs) are classified as fiber-reinforced polymer (FRP) composites. They consist of continuous glass fibers embedded in a cross-linked epoxy- or methacrylate-based resin matrix [4]. This matrix binds the fibers, transmits interfacial stress, and provides the biomechanical compliance and corrosion resistance that make these posts viable alternatives to metallic and ceramic systems [5]. Because the post is a polymeric biomaterial, its clinical retention depends not only on dentin preparation but also on compatibility between the post’s resin matrix and the luting agent [4]. Typically, this agent is a dual-cure or self-adhesive resin cement, whose monomer chemistry, degree of conversion, and micromechanical interlocking with the collagen-rich hybrid layer are critical in determining the durability of the post–cement–dentin bond [4].

Post-space preparation invariably generates a residual smear layer (SL): a 1–2 μm film of dentin debris, pulp tissue remnants, denatured collagen, and viable microorganisms [6]. This layer occludes dentinal tubule orifices, impedes resin cement interlocking, attenuates hybrid layer formation, and significantly reduces push-out bond strength (PBS) [7]. Its persistence further compromises apical seal integrity, sustaining a microenvironment conducive to microleakage and microbial survival [8]. Self-adhesive resin cements such as RelyX U200, used in the present study, contain acidic phosphate-functionalized methacrylate monomers that partially demineralize and infiltrate the smear layer and interact chemically with calcium in hydroxyapatite without a separate etching step; their bonding to radicular dentin may therefore be affected by the amount of smear layer and mineral remaining after final conditioning and by residues of the conditioning agents [9]. Effective post-space conditioning is therefore considered important for durable GFP retention, although the extent of SL removal required depends on the chemistry of the luting cement [10].

Sodium hypochlorite (NaOCl) remains the irrigant of choice in endodontics owing to its potent broad-spectrum antimicrobial activity and capacity to dissolve organic tissue; its action, however, is confined to the organic component of the SL, leaving the inorganic mineral fraction intact [11]. Ethylenediaminetetraacetic acid (EDTA) is therefore employed as a complementary final conditioning agent; its calcium-chelating action dissolves the inorganic hydroxyapatite fraction, achieving complete SL elimination and facilitating resin cement penetration into patent dentinal tubules [12]. This benefit is not without cost. EDTA exerts a pronounced demineralizing effect on radicular dentin that reduces microhardness and fracture resistance, and its intra- and peritubular erosive action—particularly when combined with NaOCl—exposes denuded collagen fibrils susceptible to enzymatic degradation and bacterial recolonization [13,14]. These recognized limitations have motivated sustained investigation into alternative post-space conditioning strategies over the past two decades.

Antimicrobial photodynamic therapy (aPDT) has emerged as a promising adjunctive modality. It photoactivates a photosensitizer (PS) with light of a specific wavelength to generate reactive oxygen species (ROS), which inflict irreversible oxidative damage to bacterial cell membranes, nucleic acids, and enzymatic machinery [15,16]. Methylene blue (MB) is among the most extensively studied photosensitizers in endodontic aPDT and demonstrates broad-spectrum bactericidal efficacy, including activity against resistant strains such as Enterococcus faecalis [17]. Although aPDT is intended principally for antimicrobial activity, it is often applied as the final step before post cementation, so its effects on the dentin substrate and on bonding are clinically relevant. Nevertheless, Ghasemi and associates reported that MB-mediated aPDT alone is insufficient for SL removal, and evidence on its specific influence on post-space SL removal and GFP PBS to radicular dentin remains scarce, warranting systematic investigation [18].

In parallel, nanotechnology has introduced superparamagnetic iron oxide nanoparticles (SPIONs) as a novel frontier in nanomedicine-based endodontic irrigation [19]. Their small size is proposed to facilitate access to dentinal tubules and anatomical irregularities poorly reached by conventional needle irrigation, and magnetically driven nanoparticle motion is proposed to agitate the irrigant and mechanically disrupt the SL [20]. Al-Mustwfi and associates reported that magnetically activated iron oxide nanoparticle irrigation was superior to EDTA in canal debridement and tubule opening [20]. However, the efficacy of SPIONs within the post space, and their influence on GFP bond strength, has not been characterized. Moreover, it has not been demonstrated whether a stationary magnet generates sufficient fluid movement or mechanical disruption to remove the SL.

Plant-derived agents have also attracted attention as low-cost alternatives to synthetic irrigants, although natural origin alone does not imply biocompatibility. Apple cider vinegar (ACV) is a naturally fermented product whose principal organic acid is acetic acid (typically about 5%), with minor amounts of malic, lactic, and citric acids; its acidic pH confers a demineralizing capacity that may dissolve the inorganic SL fraction [21,22]. In a scanning electron microscopy evaluation, Ali et al. found 5% ACV significantly more effective than EDTA in SL removal at the apical third [23]. Its efficacy as a dedicated post-space conditioning agent, and its influence on SL removal and GFP PBS, remain insufficiently characterized.

Collectively, aPDT-MB, SPIONs, and ACV each address a distinct shortcoming of the conventional NaOCl–EDTA protocol—residual microbial burden, limited tubular penetration, and iatrogenic dentin demineralization, respectively—yet none has been evaluated against the EDTA benchmark for post-space SL removal and GFP retention. These agents act through different primary mechanisms—photosensitizer-mediated oxidation intended mainly for antimicrobial activity (aPDT-MB), proposed particle-mediated mechanical action (SPIONs), and acid demineralization (ACV), compared with EDTA chelation—and were therefore compared as complete final-conditioning protocols rather than as interchangeable SL removal agents. To address this gap, this in vitro study evaluated and compared the effects of these three contemporary final post-space agents with 17% EDTA on SL removal and PBS of GFPs in post-space dentin across the cervical, middle, and apical thirds. Because all teeth first received conventional root canal treatment, including a final EDTA rinse, the study evaluated post-space treatment after a common endodontic sequence rather than replacing EDTA throughout treatment. It was hypothesized that SL removal would not differ significantly among post spaces conditioned with aPDT-MB, SPION, and ACV relative to the control, and that GFP PBS to canal dentin would remain comparable between the contemporary regimens and conventional EDTA.

## 2. Materials and Methods

Preparation of SPIONs Irrigation Solution: A 1% (*w*/*v*) SPIONs suspension was prepared by dispersing 100 mg of Fe_3_O_4_ dried powder (nominal particle size 25–50 nm according to the supplier; US Research Nanomaterials Inc., Houston, TX, USA) in 10 mL of deionized water. Ultrasonic dispersion was applied for 20 min in an ultrasonic bath (Elmasonic S 30 H, Elma Schmidtbauer GmbH, Singen, Germany; frequency 37 kHz, effective ultrasonic power 80 W) at room temperature to achieve uniform nanoparticle distribution and produce a stable colloidal suspension. Colloidal stability and particle size distribution were verified immediately after preparation by dynamic light scattering (Zetasizer Nano ZS, Malvern Panalytical, Malvern, UK) [24]. The hydrodynamic diameter (Z-average), polydispersity index (PDI), and zeta potential were measured at 25 °C in triplicate for each of three independently prepared batches. Suspensions with a PDI < 0.3 and zeta potential ≤ −10 mV were considered acceptable for use. These thresholds served only as screening criteria for the short (≤15 min) period of use, together with the absence of visible sedimentation; they do not by themselves establish long-term colloidal stability. The pH of the final suspension was measured using a calibrated digital pH meter (Mettler-Toledo, LLC, Columbus, OH, USA) [25]. Each suspension was freshly prepared, re-sonicated for 60 s in the same bath immediately before application, and used within 15 min of preparation; no visible sedimentation was observed during the 60 s application. According to the supplier’s documentation, the powder consisted of magnetite (Fe_3_O_4_) of 99.5% purity, and the certificate of analysis confirmed its single-phase composition by X-ray diffraction. The supplier designated it as superparamagnetic; this study did not independently characterize the particles’ magnetic properties or crystalline phase.

Nanoparticle Characterization by SEM/EDX: A small amount of the dry Fe_3_O_4_ powder was mounted on an aluminum stub, sputter-coated with gold in the same manner as the dentin specimens, and examined by FE-SEM (Apreo 2, Thermo Fisher Scientific, Waltham, MA, USA) at 30 kV and ×70,000. Elemental composition was analyzed at 30 kV using the EDX detector integrated with the same FE-SEM, by spectral mapping of the imaged field (768 × 512 pixels; acquisition time 304 s; average count rate ≈ 41,900 cps; ≈1.13 × 10^7^ total counts), and the summed spectrum was quantified by standardless semi-quantification; results are reported in weight percent (wt%).

**Figure 1:**
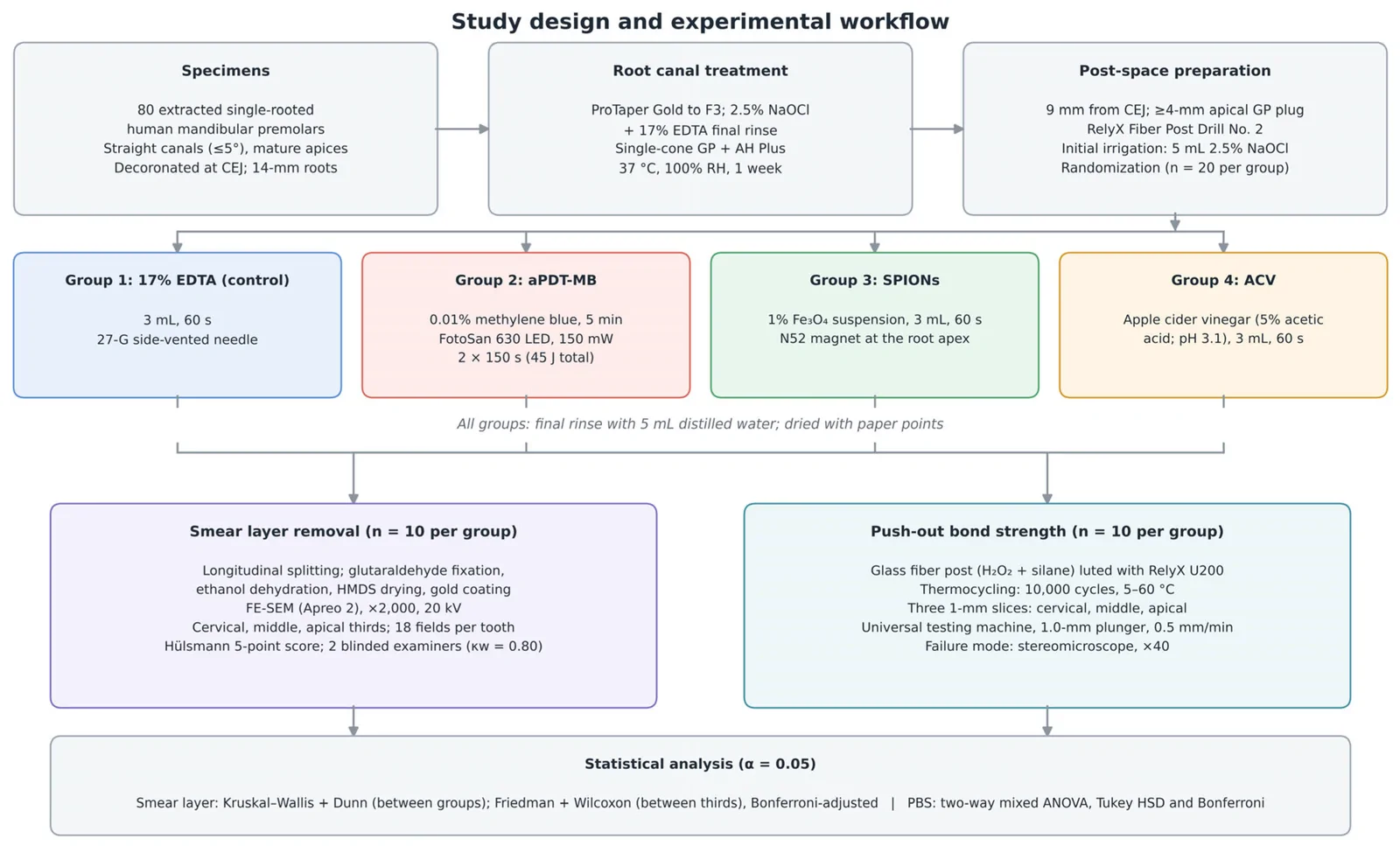
Study design and experimental workflow. Eighty single-rooted mandibular premolars received root canal treatment, including a final 17% EDTA rinse and post-space preparation, and were randomized to four final post-space treatment protocols (*n* = 20 per group). Within each group, ten teeth were allocated to SEM evaluation of smear layer removal and ten to push-out bond strength testing after glass fiber post cementation and thermocycling. aPDT-MB = methylene blue-mediated antimicrobial photodynamic therapy; SPIONs = superparamagnetic iron oxide nanoparticles; ACV = apple cider vinegar; EDTA = ethylenediaminetetraacetic acid; GP = gutta-percha; RH = relative humidity; PBS = push-out bond strength.

Sample Selection: Eighty freshly extracted single-rooted human mandibular premolars, removed for orthodontic or periodontal reasons, were recruited. Informed Consent was obtained from patients whose teeth were used in the present study, and teeth were randomized across groups. All teeth were scaled immediately after extraction to remove calculus, plaque, and soft tissue remnants using a manual periodontal scaler (Hu-Friedy, Chicago, IL, USA) and maintained in 0.1% thymol solution (Sigma-Aldrich, St. Louis, MO, USA) at 4 °C for up to three months. Teeth were eligible if they presented with fully formed apices, single straight canals verified radiographically in both projections, and no evidence of caries, fractures, prior root canal treatment, resorption, or calcifications. Roots exhibiting curvature beyond 5° per Schneider’s method were also excluded [26]. The tooth was the experimental unit. Ten teeth per group were used for each endpoint, consistent with comparable post-space push-out studies; a sensitivity analysis (G*Power 3.1; mixed-design ANOVA with four groups and three repeated root levels, α = 0.05, power = 0.80, assumed correlation among repeated measurements = 0.5) indicated that 40 teeth per endpoint were sufficient to detect a between-group effect of f = 0.45 and a protocol × root-level interaction of f = 0.25. Figure 1 summarizes the study design and experimental workflow.

Root Canal Treatment: All roots were sectioned at the cementoenamel junction to a standardized length of 14 mm using a low-speed diamond disc (Isomet 1000, Buehler, USA) under continuous water coolant. Working length was determined 1 mm coronal to the apical foramen with a size 10 K-file and verified electronically (Root ZX II, J. Morita, Japan). Canal shaping was accomplished with a rotary nickel-titanium system (ProTaper Gold, Dentsply Sirona, Switzerland) to an apical size of F3 (ISO 30, taper 0.09); with shaping files (SX, S1, S2) operated at a torque of 3.0 N·cm and speed of 300 rpm, and finishing files (F1, F2, F3) at a torque of 2.0 N·cm and speed of 250 rpm using a calibrated endodontic motor (X-Smart Plus, Dentsply Sirona, Ballaigues, Switzerland). Between instruments, canals were flushed with 5 mL of 2.5% NaOCl (Cerkamed, Warsaw, Poland) through a 27-gauge side-vented needle placed 2 mm short of working length, followed by a final rinse with 5 mL of 17% EDTA (Pulpdent, IL, USA) for 60 s. Canals were then rinsed with 5 mL of sterile saline and dried with F3 paper points. Single cone obturation was carried out with matched F3 gutta-percha and AH Plus sealer (Dentsply DeTrey, Berlin, Germany) applied to the root canal walls via a lentulo spiral. Access cavities were closed with Cavit (3M ESPE, IL, USA), and specimens were incubated at 37 °C in 100% relative humidity for one week to allow complete sealer setting [7].

Post Space Preparation: After one week, temporary restorations were removed, and gutta-percha was cleared to a depth of 9 mm from the CEJ with a Peeso reamer (Size 2, 0.70 mm; Dentsply Maillefer, Zurich, Switzerland) under irrigation, preserving a minimum 4 mm apical plug verified radiographically. The post space was then refined with the manufacturer-supplied post drill (RelyX Fiber Post Drill No. 2; 3M ESPE, IL USA) to achieve passive post seating. Because the post drill enlarged the canal beyond its instrumented dimensions along the full post-space length, the dentin exposed to EDTA during root canal treatment was removed and a fresh smear layer was created on all post-space walls before the experimental protocols. Post-space diameter and taper were standardized by the drill, and the coronal and apical radii of each push-out slice were measured; residual dentin thickness was not measured. A single calibrated operator completed all preparations [27].

Experimental Groups and Post Space Conditioning Protocols: Before group allocation, all post spaces received an initial irrigation with 5 mL of 2.5% NaOCl through a 27-gauge side-vented needle. Specimens were subsequently divided into four equal groups (n = 20) via computer-generated randomization (Research Randomizer, Version 4.0). Within each group, ten teeth were then allocated to SEM evaluation and ten to push-out testing using a second computer-generated list. No specimen was lost or excluded, and all 80 teeth contributed to the analysis.

Group 1: Control-17% EDTA: 3 mL of 17% EDTA (Pulpdent, USA) was delivered through a 27-gauge side-vented needle and retained for 60 s.

Group 2: aPDT-MB: 1 mL of 0.01% methylene blue was introduced into the canal via a 27-gauge needle and left undisturbed for 5 min to facilitate dye penetration. Canals were dried with paper points before irradiation. Irradiation was performed with a light-emitting diode (LED) photodynamic device (FotoSan 630, CMS Dental, Copenhagen, Denmark; emission spectrum 620–640 nm) fitted with a 1 mm diameter endodontic light tip (emitting area 0.00785 cm^2^), which was inserted to 1 mm short of the post-space floor (8 mm from the canal orifice), as the apical gutta-percha plug remained in place. The output measured at the tip was 150 mW, verified with a calibrated optical power meter (PM100D with S142C sensor, Thorlabs, USA), in continuous mode, corresponding to an irradiance of 19.1 W/cm^2^ for the stated emitting area. The tip was withdrawn in an apical-to-cervical spiral motion. Two 150 s irradiation cycles were separated by a 150 s rest interval; each cycle delivered 22.5 J (radiant exposure 2866 J/cm^2^), giving a total of 45 J (5732 J/cm^2^) per specimen. The total aPDT-MB treatment time was therefore 12.5 min (5 min pre-irradiation followed by 7.5 min of irradiation and rest), compared with 60 s for the other protocols [28].

Group 3: SPIONs. 3 mL of the prepared SPION suspension was delivered into the post space for 60 s. Simultaneously, a neodymium (N52) disc magnet (5 mm diameter × 3 mm thickness; surface field strength 0.3 T) was held stationary in contact with the external root surface at the apex, with its flat face perpendicular to the long axis of the root, approximately 5 mm from the floor of the post space (14 mm root; 9 mm post space), for the 60 s application to attract the nanoparticles apically [19].

Group 4: ACV. 3 mL of undiluted commercial apple cider vinegar (Aldurra Inc., Damascus, Syria; labeled acidity 5% acetic acid; measured pH 3.1) was introduced via a 27-gauge needle and retained for 60 s.

All groups received a final rinse with 5 mL distilled water, followed by drying with paper points. A single trained operator performed all procedures under uniform conditions.

Scanning Electron Microscopy (SEM) evaluation of Smear Layer Removal: Ten specimens from each experimental group underwent scanning electron microscopy (SEM) analysis to evaluate smear layer removal following application of final conditioning protocols. Roots were longitudinally sectioned by creating two opposing grooves along the buccal and lingual surfaces using a chisel and mallet, yielding matched halves that exposed the root canal walls. Sectioned halves were rinsed with phosphate-buffered saline (PBS), fixed in 4% glutaraldehyde in 0.1 mol/L sodium cacodylate buffer (BDH, London, England) at pH 7.4, and maintained at 4 °C overnight. Dehydration was performed through an ascending ethanol series (Honeywell, Berlin, Germany): 30%, 50%, and 70% for 10 min each, followed by 90% and 100% for 20 min each. Chemical drying was completed by immersion in hexamethyldisilazane (HMDS; Merck, Berlin, Germany) for 5 min, after which specimens were air-dried at room temperature. Dried specimens were mounted on aluminum stubs, sputter-coated with a uniform gold layer to enhance electrical conductivity, and examined under a field emission scanning electron microscope (FE-SEM; Apreo 2, Thermo Fisher Scientific, USA). Each root was evaluated at three standardized regions along the 9 mm prepared post space: the cervical third (0–3 mm), middle third (3–6 mm), and apical third (6–9 mm) from the canal orifice [29]. Scoring was performed on micrographs acquired at a standardized magnification of ×10,000 (10–30 kV); the magnification, accelerating voltage, and scale bar of each micrograph are recorded in its instrument data bar. In each third of each root half, three non-overlapping fields were recorded at the center of the third at predefined positions located at low magnification before the surface was viewed at the scoring magnification, giving 18 fields per tooth. Smear layer removal was scored using the Hülsmann scoring index to evaluate the efficacy of each conditioning protocol [30].

Score 1 indicated no SL with fully patent dentinal tubules.

Score 2 indicated a small amount of SL with some dentinal tubules open.

Score 3 indicated homogeneous SL covering the canal wall with only a few dentinal tubules open.

Score 4 indicated homogeneous SL covering the complete canal wall with no open dentinal tubules.

Score 5 indicated heavy, non-homogeneous SL covering the complete canal walls.

Specimens were coded, and two calibrated examiners, both blinded to group allocation, scored all fields independently. Interexaminer agreement was assessed using linearly weighted Cohen’s kappa (κw = 0.80), calculated on all 720 field scores (18 fields × 40 teeth), and disagreements were resolved by consensus. For each tooth and root third, the field scores from both root halves and both examiners were averaged (12 integer ratings per third: three fields × two root halves × two examiners) to give a tooth-level score; group results are reported as the median and interquartile range (25th–75th percentiles) of the ten tooth-level scores. Because tooth-level scores are means of integer ratings, they can take non-integer values; the interquartile range describes the spread of the ten tooth-level scores and was not averaged further.

Glass Fiber Post Cementation: Following post space conditioning, glass fiber posts (No. 2, 1.5 mm diameter; RelyX Fiber Post, 3M ESPE, IL, USA) were trialed in the post space of 10 samples from each group to verify passive fit. Post surfaces were conditioned with 24% hydrogen peroxide for 60 s, rinsed thoroughly with distilled water for 60 s to remove residual peroxide, air-dried, and treated with a methacryloxypropyltrimethoxysilane coupling agent (Monobond Plus, Ivoclar Vivadent, Vaduz, Liechtenstein) for 60 s, followed by air evaporation. This post pretreatment was applied identically in all groups as a standardized experimental factor (see Section 4). Cementation was performed using a self-adhesive dual-cure resin cement (RelyX U200, 3M ESPE, IL USA), mixed per manufacturer instructions, and was applied to the post surface and canal walls via a lentulo spiral at low speed. Posts were seated under finger pressure, excess cement was removed, and light curing was performed at the canal orifice for 40 s at ≥1000 mW/cm^2^ (Elipar S10, 3M ESPE, IL USA). All specimens were stored at 37 °C in 100% relative humidity for 24 h before mechanical testing [31].

Thermocycling: After the 24-h setting interval, the bonded specimens underwent a thermal fatigue regimen intended to replicate the aging the post–cement–dentin complex experiences intraorally (THE-1100 thermocycler; SD Mechatronik, Feldkirchen-Westerham, Berlin, Germany). Each specimen was cycled 10,000 times between two temperature-controlled reservoirs set at 5 °C and 60 °C; immersion in each reservoir lasted 45 s, and the interval required to shuttle specimens between them was fixed at 5 s. The samples were then held in distilled water at 37 °C pending mechanical evaluation [32]. This extended regimen modifies the thermocycling procedure described in ISO/TS 11,405:2015 [32] (500 cycles between 5 °C and 55 °C, dwell time ≥ 20 s) by increasing the number of cycles and the upper bath temperature; therefore, it is not presented as compliant with that specification.

Slicing of the Specimens for the Push-Out Test: Each root was sectioned perpendicular to its long axis into three slices using a diamond-coated slow-speed band saw (Hager and Meisinger GmbH, Neuss, Germany) operated at 150 rpm under continuous water cooling. Sectioning commenced 1 mm apical to the CEJ, yielding 1 mm thick slices sampled at depths of 2 mm (cervical), 5 mm (middle), and 8 mm (apical). Slice thickness was confirmed using a digital caliper (±0.01 mm), and each section was marked on its apical surface for orientation [33].

Push-Out Bond Strength: Each slice was positioned on a metal support with a central aperture exceeding the post diameter, ensuring that the applied load was directed solely onto the post. A universal testing machine (Instron 3345, Instron Corp., Chicago, IL, USA) fitted with a flat-ended cylindrical plunger (1.0 mm diameter) delivered compressive force in the apico-coronal direction at a crosshead speed of 0.5 mm/min until debonding occurred. Before loading, the plunger was centered on the post under ×20 magnification; the post diameter at the apical slice (≈1.1 mm) exceeded the 1.0 mm plunger diameter, and the absence of plunger contact with cement or dentin was confirmed by inspecting each slice after testing. Push-out bond strength (PBS, MPa) was calculated by dividing the load at failure (F, N) by the bonded surface area (A, mm^2^) (Equation (1)):

(1) $$\mathrm{PBS}=\frac{F}{A}$$

A was calculated for the cement–dentin interface as the lateral surface area of a truncated cone (Equation (2)):

(2) $$A=\pi \left(R+r\right)\sqrt{{\left(R-r\right)}^{2}+h^{2}}$$

where R and r are the coronal and apical radii of the post space of each slice, measured under the stereomicroscope at ×40, and h is the slice thickness measured with a digital caliper. No specimen debonded before testing, so no slice was excluded on this basis. Slices with cohesive failure were retained in the analysis; for these, the calculated value represents the nominal debonding stress rather than a direct estimate of interfacial bond strength.

Failure Mode Analysis: Debonded sections were examined under a stereomicroscope at ×40 magnification (Nikon SMZ800, Tokyo, Japan) and classified as adhesive at the post–cement interface, adhesive at the cement–dentin interface, cohesive (within cement, dentin, or post, where reliably identifiable), or admixed (adhesive and cohesive features on the same slice). Failure modes were recorded for every slice (30 per group; 10 per root third) by an examiner blinded to group allocation.

Statistical Analysis: Data were analyzed using SPSS Version 26.0 (IBM Corp., Chicago, IL, USA) at a significance threshold of *p* < 0.05. The tooth was the unit of analysis. PBS data were analyzed using a two-way mixed ANOVA with conditioning protocol (four levels) as the between-subjects factor and root level (cervical, middle, apical) as the within-subjects factor. Normality of residuals was assessed with the Shapiro–Wilk test and Q–Q plots, homogeneity of variances with Levene’s test at each root level, homogeneity of covariance matrices with Box’s M test (evaluated at α = 0.001, given its sensitivity), and sphericity with Mauchly’s test for the within-subjects error term. Because the root-level main effect and the protocol × root-level interaction share this error term, a single Mauchly’s test applies to both effects, and the Greenhouse–Geisser correction was applied to the degrees of freedom of both effects when sphericity was violated. Effect sizes are reported as partial eta squared (ηp^2^). Because the protocol × root-level interaction was significant, simple effects were analyzed: protocols were compared at each root level using one-way ANOVA with Tukey’s test, and root levels were compared within each protocol using paired comparisons with Bonferroni adjustment. Tukey-adjusted 95% confidence intervals (CIs) are reported for key mean differences. SL scores, being ordinal, were compared among protocols at each root third using the Kruskal–Wallis test followed by Dunn’s test with Bonferroni adjustment, and among root thirds within each protocol using the Friedman test followed by Wilcoxon signed-rank tests with Bonferroni adjustment (adjusted α = 0.017). Effect sizes were epsilon-squared (ε^2^) for the Kruskal–Wallis tests and Kendall’s W for the Friedman tests. Group medians and 25th–75th percentiles of the tooth-level SL scores were computed as interpolated (grouped) values in SPSS. Between-group comparisons at each root third involved independent teeth, whereas the Friedman test accounted for the repeated root thirds within each tooth. Failure modes are reported descriptively as counts.

## 3. Results

**Figure 2:**
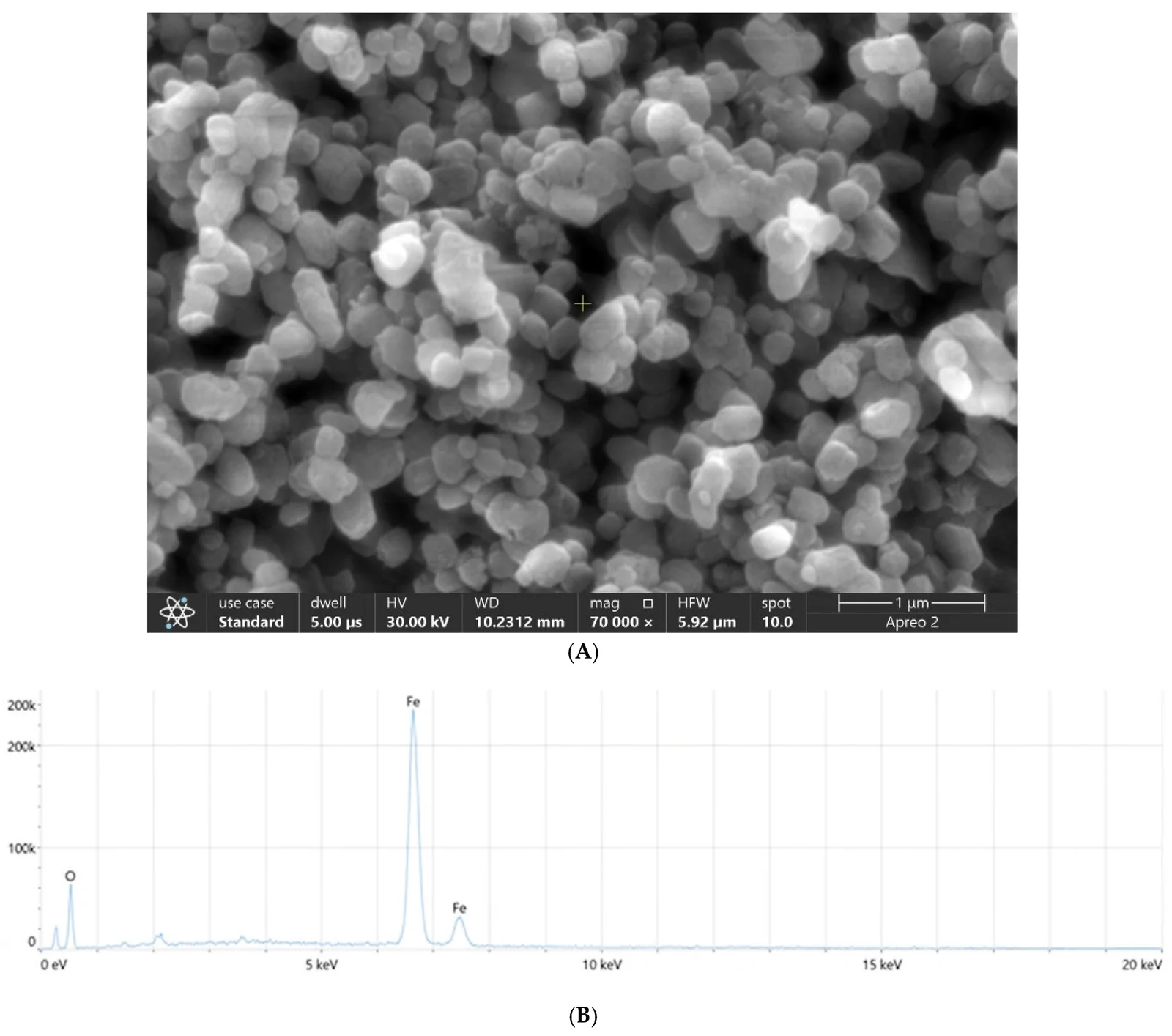
(**A**) SEM micrograph of iron oxide particles (30 kV, ×70,000; scale bar = 1 µm) showing quasi-spherical to sub-rounded, near-equiaxed particles with smooth, well-defined boundaries, interparticle necking, and irregular agglomerates with interparticle voids. (**B**) Summed EDX spectrum from a 304 s map acquired from the field shown in A, showing Fe (54.8 wt%) and O (44.2 wt%) as the principal elements; minor unlabelled peaks at ~0.28 and ~2.1 keV correspond to C and the Au coating. EDX values are semi-quantitative and do not identify the iron oxide phase.

Nanoparticle Characterization: DLS of the freshly prepared suspension gave a Z-average hydrodynamic diameter of 78.4 ± 6.2 nm, a PDI of 0.21 ± 0.03, and a zeta potential of −18.6 ± 1.4 mV (mean ± SD, n = 3); the suspension pH was 6.8. SEM showed quasi-spherical particles with an apparent size of approximately 100–250 nm; many coalesced or necked into agglomerates. These dimensions exceed the supplier’s nominal 25–50 nm range; because this magnification could not reliably resolve individual primary crystallites, the SEM features most likely represent coalesced particles and agglomerates, whereas the DLS diameter reflects hydrated aggregates in suspension. Transmission electron microscopy would be required to determine primary particle size. EDX of the same field showed a dominant Fe Kα peak (~6.4 keV) with a secondary Fe Kβ signal (~7.05 keV) and a distinct O Kα peak (~0.52 keV); Fe and O were the principal elements detected across 0–20 keV; minor unlabelled peaks near 0.28 keV and 2.1 keV are consistent with carbon and the gold sputter coating, respectively (Figure 2). Standardless semi-quantitative analysis gave Fe 54.8 wt% and O 44.2 wt%; these values deviate from the stoichiometric composition of Fe_3_O_4_ (72.4 and 27.6 wt%), most likely because light-element quantification on an irregular, gold-coated powder agglomerate is unreliable, and were not used to infer the phase. EDX confirms elemental composition but does not identify the iron-oxide phase or establish superparamagnetic behavior.

**Figure 3:**
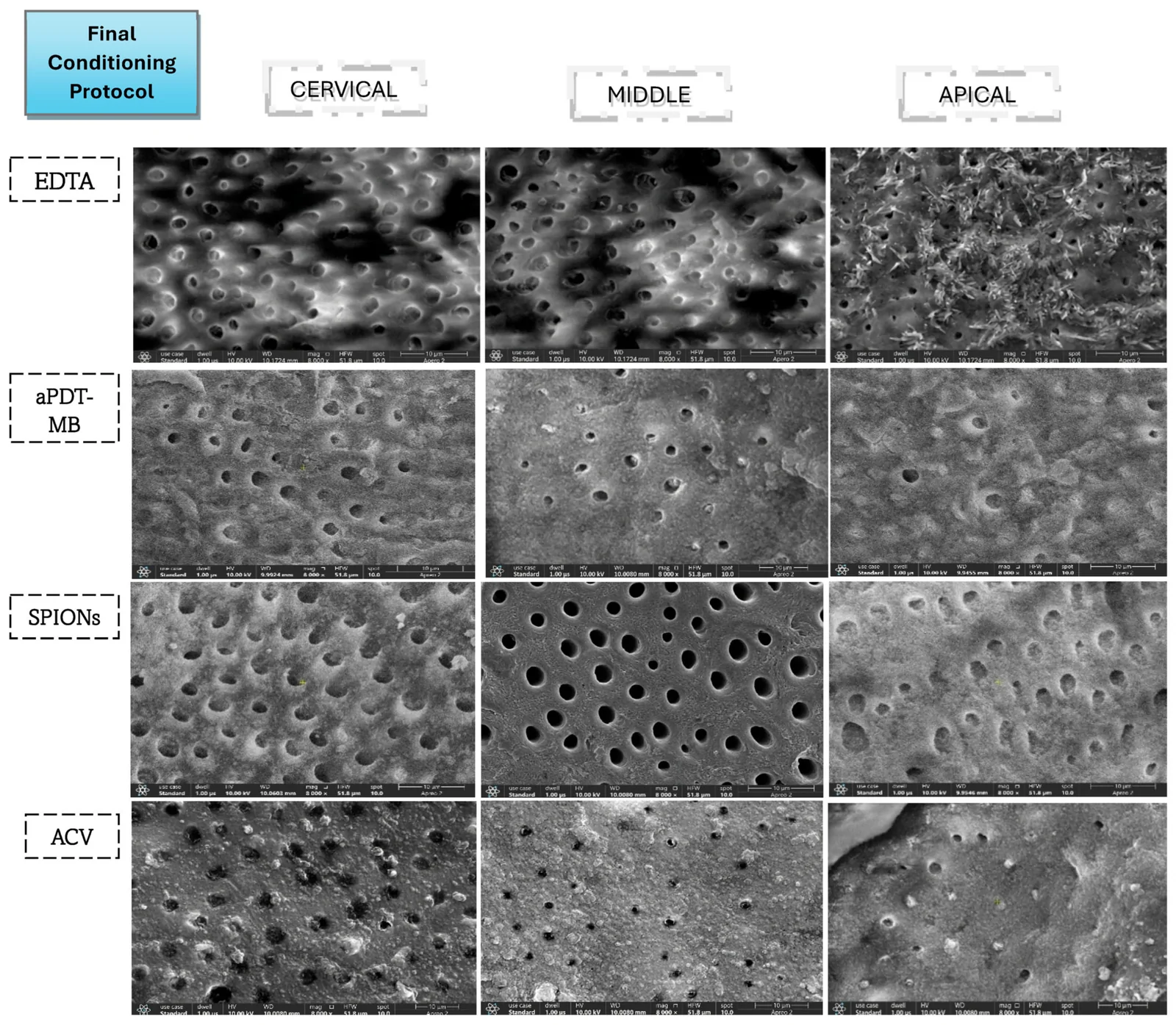
Representative scanning electron micrographs of post-space canal walls following the four final conditioning protocols, arranged by root third. Rows correspond to the conditioning groups (Group 1: EDTA; Group 2: aPDT-MB; Group 3: SPIONs; Group 4: ACV) and columns to the cervical, middle, and apical thirds. Open, patent dentinal tubule orifices with clean peritubular dentin indicate effective smear layer removal, whereas occluded tubules with a confluent amorphous smear layer indicate reduced removal efficacy. Smear layer retention increased from the cervical to the apical third in Groups 1, 2, and 4, whereas no significant regional difference was detected in Group 3. Each panel shows the scored field closest to the group median score for that third (Apreo 2, Thermo Fisher Scientific). All micrographs were acquired at ×10,000; the accelerating voltage and scale bar of each panel are given in its instrument data bar. ACV = apple cider vinegar; aPDT-MB = antimicrobial photodynamic therapy with methylene blue; EDTA = ethylenediaminetetraacetic acid; SPIONs = superparamagnetic iron oxide nanoparticles.

Smear layer removal: Table 1 presents the smear layer (SL) removal efficacy of the four conditioning protocols across the three root thirds. Values are medians (interquartile range, 25th–75th percentiles) of the ten tooth-level scores per group; descriptive statistics and omnibus test statistics with effect sizes are given in Supplementary Tables S2 and S3. The cervical third of Group 3 (NaOCl + SPIONs) recorded the lowest median score of any group and root third [median (IQR) 1.20 (1.08–1.31)], whereas the apical third of Group 2 (NaOCl + aPDT-MB) showed the least effective removal [3.54 (3.30–3.88)]. Intergroup comparison (Kruskal–Wallis: cervical H(3) = 32.1, middle H(3) = 29.3, apical H(3) = 29.9; all *p* < 0.001) revealed that Group 1 [EDTA; cervical 1.30 (1.06–1.35); middle 1.35 (1.03–1.55); apical 2.40 (2.36–2.77)] and Group 3 [cervical 1.20 (1.08–1.31); middle 1.25 (1.13–1.49); apical 1.32 (1.10–1.65)] did not differ significantly at the cervical and middle thirds (Dunn–Bonferroni *p* > 0.05) but differed significantly at the apical third (*p* < 0.05). Group 4 [NaOCl + ACV; cervical 1.93 (1.80–2.07); middle 2.10 (1.72–2.28); apical 3.06 (2.81–3.25)] achieved significantly greater SL removal than Group 2 [cervical 2.60 (2.41–2.83); middle 2.72 (2.51–2.91); apical 3.54 (3.30–3.88)] (*p* < 0.05). Intragroup comparison (Friedman test with Bonferroni-adjusted Wilcoxon signed-rank tests) showed that, in Groups 1, 2, and 4 (χ^2^(2) = 15.80, 20.00, and 18.20, respectively; all *p* < 0.001), the cervical and middle thirds did not differ significantly (*p* > 0.05), whereas both had significantly lower scores than the apical third (*p* < 0.05); in Group 3, no significant difference was detected among the three thirds (χ^2^(2) = 3.80, *p* = 0.150) (Figure 3).

**Figure 4:**
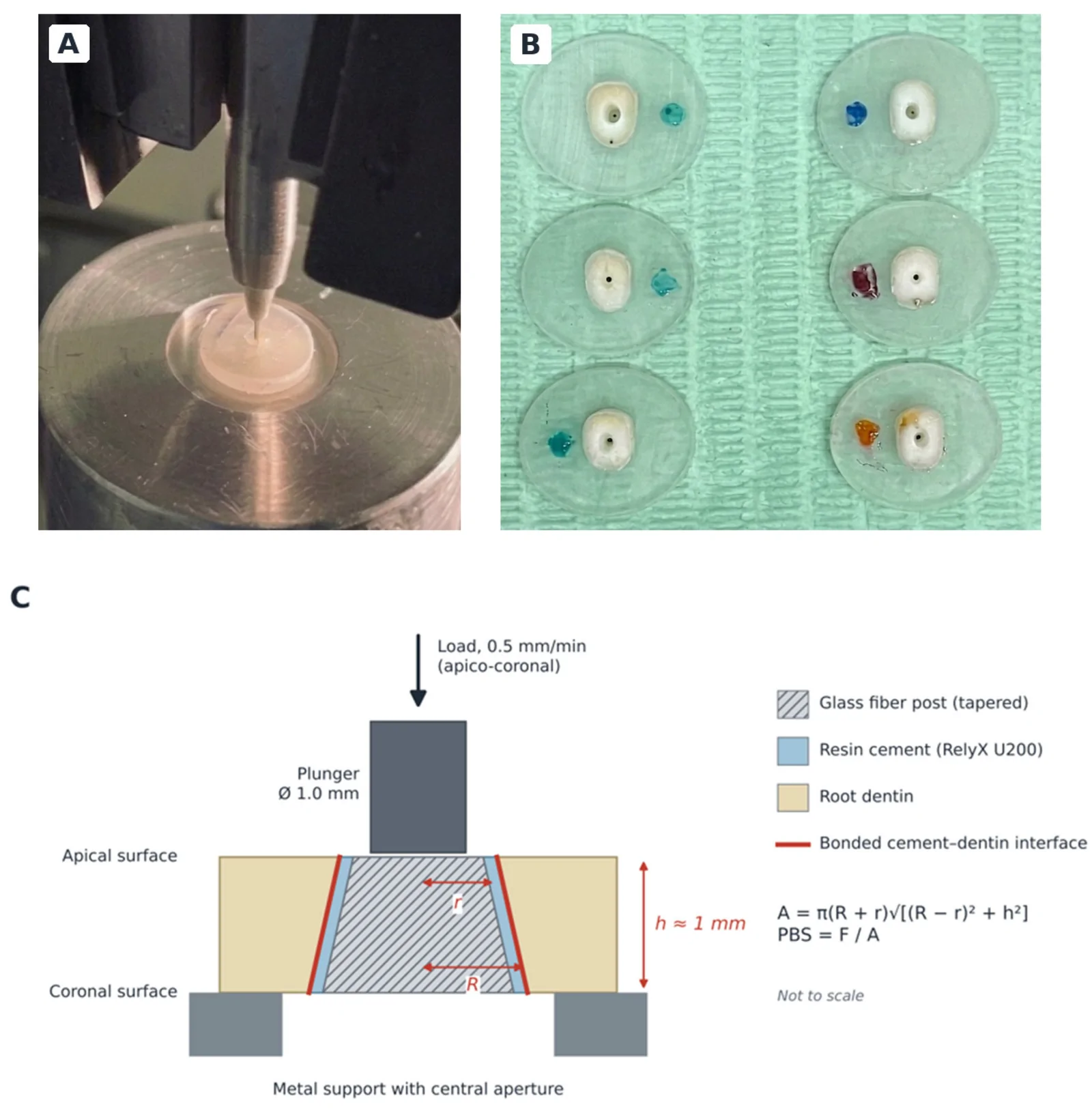
Push-out bond strength testing. (**A**) Representative specimen mounted on the universal testing machine, showing the 1.0-mm flat-ended cylindrical plunger centered over the glass fiber post of a 1-mm-thick root slice supported on a metal jig with a central aperture, with load applied apico-coronally. (**B**) Representative sectioned root slices after testing, with the dislodged post segments alongside each slice. (**C**) Schematic of the test configuration (not to scale): the slice is placed with its apical surface facing the 1.0-mm plunger on a support whose aperture exceeds the coronal post diameter, and load is applied in the apico-coronal direction. R and r are the coronal and apical post-space radii, and h is the slice thickness used to calculate the bonded cement–dentin area (Equation (2)). GFP = glass fiber post.

Push-out bond strength: Table 2 presents the PBS values of GFPs following different root canal conditioning protocols. The mixed ANOVA showed significant main effects of conditioning protocol (F(3, 36) = 176.4, *p* < 0.001, ηp^2^ = 0.94) and root level (Greenhouse–Geisser corrected, ε = 0.739: F(1.5, 53.2) = 276.0, *p* < 0.001, ηp^2^ = 0.89) and a significant protocol × root-level interaction (Greenhouse–Geisser corrected: F(4.4, 53.2) = 33.1, *p* < 0.001, ηp^2^ = 0.73); Box’s M test indicated no significant heterogeneity of the covariance matrices across protocols (Box’s M = 15.75, F(18, 4579.7) = 0.74, *p* = 0.77); sphericity, assessed with a single Mauchly’s test for the within-subjects error term shared by the root-level main effect and the protocol × root-level interaction, was violated (Mauchly W = 0.65, χ^2^(2) = 15.08, *p* < 0.001), and the Greenhouse–Geisser correction (ε = 0.739) was therefore applied to both effects; residuals were normally distributed (Shapiro–Wilk *p* = 0.37), and variances were homogeneous at each level (Levene *p* ≥ 0.36). Protocols differed significantly at each root level (cervical: F(3, 36) = 113.6; middle: F(3, 36) = 118.7; apical: F(3, 36) = 206.9; all *p* < 0.001). Group 3 (NaOCl + SPIONs) recorded the highest bond strength at the cervical third (11.20 ± 0.84 MPa), while the lowest bond strength across all groups and thirds was observed at the apical third of Group 2 (NaOCl + aPDT-MB) (3.90 ± 0.52 MPa). Intergroup comparison revealed that Group 1 (NaOCl + EDTA) (Cervical: 10.90 ± 0.81 MPa; Middle: 10.75 ± 0.77 MPa; Apical: 7.20 ± 0.61 MPa) and Group 3 (Cervical: 11.20 ± 0.84 MPa; Middle: 10.95 ± 0.79 MPa; Apical: 10.70 ± 0.76 MPa) did not differ significantly at the cervical (mean difference 0.30 MPa; 95% CI −0.58 to 1.18; Tukey-adjusted *p* = 0.794) or middle third (0.20 MPa; 95% CI −0.68 to 1.08; *p* = 0.927), whereas Group 3 exceeded Group 1 at the apical third (3.50 MPa; 95% CI 2.75 to 4.25; *p* < 0.001). All pairwise comparisons, with Tukey-adjusted *p*-values and 95% CIs, are given in Supplementary Table S1. Group 4 (NaOCl + ACV) (cervical: 7.90 ± 0.65 MPa, middle: 7.60 ± 0.71 MPa, apical: 6.10 ± 0.58 MPa) demonstrated significantly higher bond strength than Group 2 (Cervical: 6.10 ± 0.58 MPa; Middle: 5.80 ± 0.63 MPa; Apical: 3.90 ± 0.52 MPa) at all root thirds (mean differences 1.80–2.20 MPa; lower 95% CI limits ≥ 0.92 MPa; all *p* < 0.001) but lower bond strength than Groups 1 and 3 (*p* < 0.05). Intragroup comparisons showed that in Groups 1, 2, and 4, cervical and middle third bond strengths did not differ significantly (*p* > 0.05), but both differed significantly from their respective apical third values (*p* < 0.05). In contrast, Group 3 exhibited no statistically significant difference across all three root thirds (*p* > 0.05) (Figure 4).

**Figure 5:**
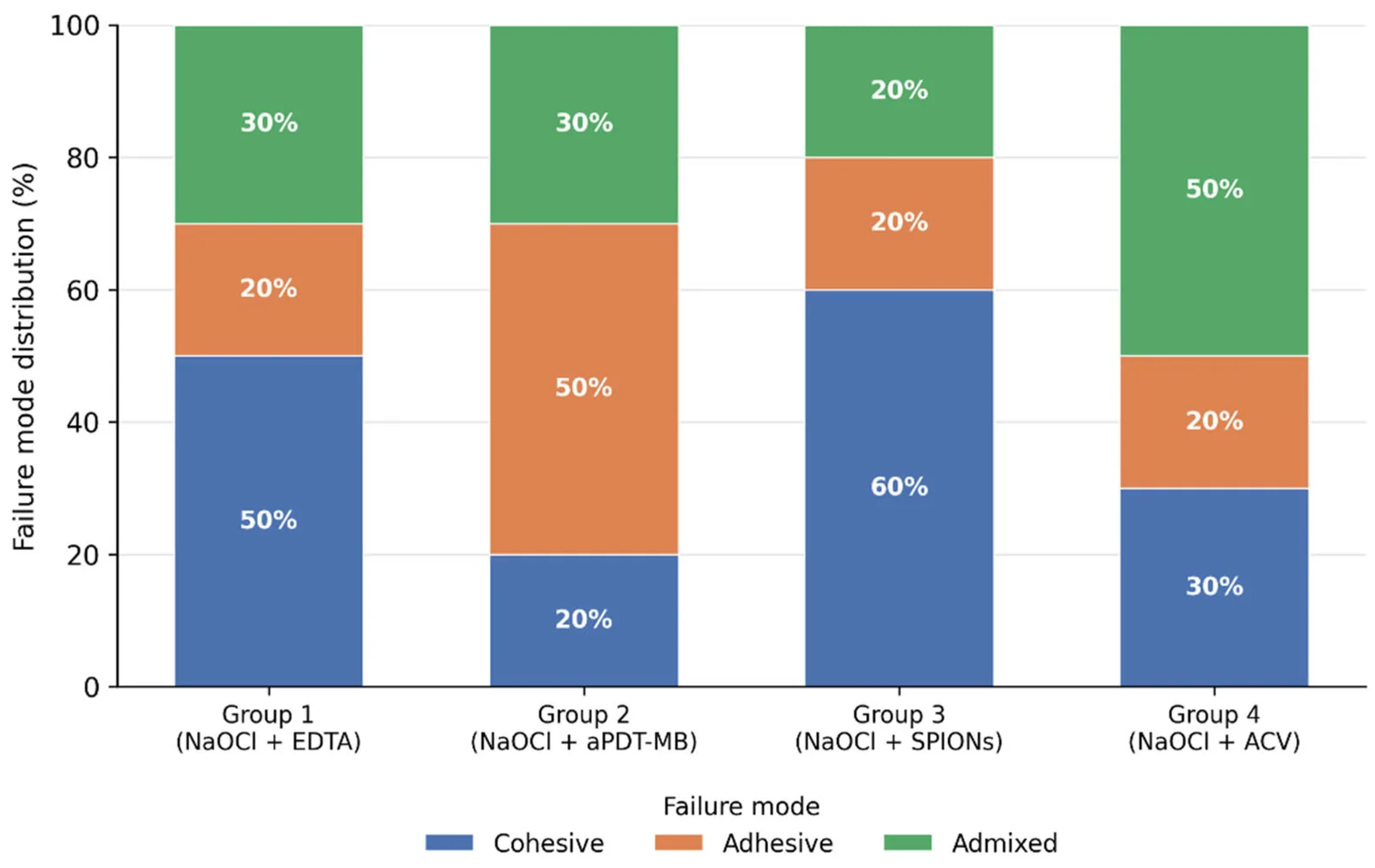
Distribution of failure modes (%) among the four experimental groups following push-out bond strength testing (denominator: 30 slices per group, 10 per root third; counts by root third are given in Table 3).

Failure mode analysis: Failure-mode counts by protocol and root third are given in Table 3, and the corresponding percentages in Figure 5 (denominator: 30 slices per group; 10 per root third). Cohesive failures were most frequent in the SPION (18 of 30) and EDTA (15 of 30) groups; admixed failures were most frequent in the ACV group (15 of 30); and adhesive failures, predominantly at the cement–dentin interface, were most frequent in the aPDT-MB group (15 of 30, of which 12 were at the cement–dentin interface). Across the four groups, post–cement adhesive failures were infrequent (9 of 120 slices), and cohesive failures occurred mainly within dentin. In every group, adhesive failures were most frequent in the apical third (Figure 5).

## 4. Discussion

This study tested the null hypotheses that post-space conditioning with aPDT-MB, SPIONs, and ACV would yield SL removal and PBS outcomes comparable to the NaOCl + EDTA control. The findings partially refuted both hypotheses: no statistically significant difference was detected between NaOCl + SPIONs and the control at the cervical and middle thirds (cervical SL median 1.20 [IQR 1.08–1.31] vs. 1.30 [1.06–1.35]). At the apical third, however, SPIONs (1.32 [1.10–1.65]) significantly outperformed EDTA (2.40 [2.36–2.77]; *p* < 0.05), and within the SPION group, no regional differences were detected, whereas SL retention increased apically in the control. By contrast, the aPDT-MB and ACV groups performed significantly below the control across all root thirds, including the readily accessible cervical region. Because no formal equivalence analysis was performed, the absence of a significant difference at the cervical and middle thirds should not be interpreted as equivalence; the 95% CIs for the PBS differences (−0.58 to 1.18 and −0.68 to 1.08 MPa) indicate that differences of about 1 MPa cannot be excluded. The PBS test was selected for its established methodological advantages, including straightforward specimen preparation via standardized horizontal root sectioning, minimal operator-dependent variability, and well-documented reproducibility across comparable investigations.

The integrity of the post–dentin adhesive interface is fundamentally contingent on adequate SL management, as a residual smear layer occludes dentinal tubules, prevents resin tag penetration, and substantially undermines bond performance. Among all experimental groups, NaOCl + SPIONs and NaOCl + EDTA showed the lowest SL scores and the highest PBS values, without significant differences between them at the cervical and middle thirds. SPIONs’ performance is consistent with observations by Al-Mustwfi and colleagues, who showed that magnetically activated iron oxide nanoparticle suspensions combined with ultrasonic potentiation achieved optimal debris and smear layer elimination throughout the root canal [20]. One proposed explanation is that magnetically driven nanoparticle motion agitates the irrigant and mechanically disrupts the smear layer. However, the study used a stationary magnet, did not measure fluid flow, and did not include a non-magnetized nanoparticle control; therefore, the relative contributions of the suspension itself (including its pH and particle abrasion) and the magnetic field cannot be determined, and this mechanism remains hypothetical [34,35]. Residual iron oxide particles could also remain on the dentin surface after rinsing and may influence the resin cement-substrate interaction even where SEM shows open tubules; elemental mapping of treated dentin (e.g., by EDX) would help clarify this. Likewise, the NaOCl + EDTA combination produced satisfactory outcomes at the cervical and middle root thirds, consistent with prior laboratory evidence [36]. This aligns with the well-characterized chelating activity of EDTA, which facilitates smear layer removal by binding calcium ions (Ca^2+^) from hydroxyapatite with its carboxylate and amine groups, forming soluble calcium–EDTA complexes [37]. This reaction selectively demineralizes the inorganic phase of both the smear layer and the peritubular dentin, effectively opening the dentinal tubules and exposing the underlying collagen matrix [38]. The comparatively diminished SL removal efficacy observed at the apical third in the EDTA group is consistent with the well-recognized limitation of conventional needle irrigation in achieving adequate irrigant delivery to the apical region. In contrast, SPIONs performed better at this level; whether this reflects magnetically enhanced irrigant penetration, as hypothesized above, requires confirmation with appropriate controls [34].

ACV-treated specimens exhibited intermediate performance, surpassing aPDT-MB across all root thirds while remaining inferior to EDTA and SPIONs. The principal acid in ACV is acetic acid, accompanied by smaller amounts of malic, lactic, and citric acids [22]. These weak organic acids are thought to remove the inorganic smear layer mainly by acid demineralization of hydroxyapatite, with a minor contribution from calcium complexation by citrate and malate [39]. Such conditioning has been reported to favor resin tag formation and bond strength with resin-based luting cements, although resin tags were not assessed in the present study [40]. The ACV used here had a measured pH of 3.1 and a labeled acetic acid content of 5%; its titratable acidity and full organic-acid composition were not measured, limiting comparison with studies that used other vinegar formulations. A laboratory-based analysis by Moness Ali and associates reported that 5% ACV exerts its greatest SL removal activity in the apical third, although its overall efficacy remained below that of EDTA across the full canal length [23]. This contrasts with the present analysis; therefore, further studies are needed to confirm these findings. In addition, ACV composition, acidity, and microbial content vary by production method, and unpasteurized vinegars may contain viable acetic acid bacteria and yeasts. The microbial content of the product used here was not tested; clinical use would therefore require a sterile, standardized preparation.

In contrast, aPDT-MB yielded the lowest SL removal efficiency and bond strength values across all root thirds. Because aPDT is intended principally for antimicrobial effects, which were not assessed here, this finding should not be interpreted as evidence that the technique is ineffective for its principal indication. This inferior performance may relate to methylene blue’s cationic charge, which has a high affinity for anionic substrates in radicular dentin—specifically calcium and phosphate ions—promoting precipitate formation on the canal walls and thereby compromising cement–dentin bond strength [41]. Furthermore, residual methylene blue on the canal wall may interfere with adhesive adaptation at the dentinal interface [42]. Consistent with this, SEM showed greater SL retention after aPDT-MB at the group level. Because SEM and push-out outcomes were obtained from different teeth, we cannot establish their relationship at the specimen level. Moreover, aPDT-MB involved a longer treatment (12.5 min vs. 60 s) and a different sequence (dye application, drying, and irradiation), and no dye-only or light-only controls were included, so the separate contributions of the dye, the light, and the treatment duration cannot be distinguished.

The failure-mode distribution paralleled the bond strength ranking. Cohesive failures predominated in the EDTA and SPION groups; however, cohesive failure in push-out testing can arise from non-uniform stress distribution and substrate defects and does not by itself demonstrate superior bonding. When failure is cohesive, the measured value reflects the strength of the failed substrate rather than the interface, limiting its interpretation as interfacial bond strength. The predominance of admixed failures after ACV and of cement–dentin adhesive failures after aPDT-MB may reflect less effective SL removal and possible residual photosensitizer, respectively. However, these explanations were not tested directly [43].

From a biomaterials standpoint, these findings indicate that smear layer management is only one determinant of post-space bond durability; the chemistry of the polymeric substrates involved is equally critical. The self-adhesive dual-cure resin cement luting the GFPs relies on acidic functional monomers that must simultaneously interact with residual mineral, infiltrate the exposed collagen network, and polymerize into a dimensionally stable matrix; any smear layer or surface contaminant that impedes monomer diffusion may compromise the degree of conversion and, consequently, the mechanical integrity of the resulting hybrid layer. Because GFP is a fiber-reinforced polymer composite, its retention also depends on micromechanical and chemical continuity between the post’s resin matrix, the resin cement, and the collagen-reinforced dentin, forming a tripartite polymeric biomaterial interface whose weakest link dictates overall performance. Greater tubule opening does not necessarily produce stronger bonding with every self-adhesive resin cement; substrate moisture, residual chemicals, the degree of demineralization, polymerization conditions, and cement viscosity may all influence retention. Moreover, the push-out test measures the response of the whole bonded assembly—fiber post, silane, resin cement, and dentin—rather than the isolated adhesion of cement to dentin [44]. The fiber posts were pretreated identically in all groups with hydrogen peroxide and silane; hydrogen peroxide removes the superficial epoxy resin and exposes the glass fibers for silanization [45], although the 60 s application used here was shorter than the protocols evaluated in that study, and this standardized pretreatment may also have influenced the post–cement interface. More broadly, irrigation and activation strategies increasingly determine how dentin substrates respond to subsequent dentin–material interactions [46]. The present study did not measure collagen preservation, surface wettability, resin-tag formation, or degree of conversion; whether NaOCl + SPIONs favor this biomaterial continuum beyond SL removal therefore remains a hypothesis for future work.

Several inherent limitations of this in vitro design warrant acknowledgment. The absence of biological fluids, masticatory loading, and mechanical fatigue precludes direct clinical extrapolation. Thermocycling reproduces aging only partially, and laboratory push-out strength should not be equated with long-term clinical retention. Standardized extracted teeth do not reflect the anatomical heterogeneity of clinical root canal systems, which may influence photosensitizer penetration depth and regional bond strength outcomes. Furthermore, fixed irrigant concentrations precluded dose–response analysis, and the present methodology did not include nanoscale ultrastructural characterization of conditioned radicular dentin surfaces by atomic force microscopy. In addition, treatment durations differed among protocols (12.5 min for aPDT-MB vs. 60 s for the other agents), and the study included no dye-only, light-only, or non-magnetized nanoparticle controls, so the separate effects of the photosensitizer, light, and magnetic field cannot be isolated. Agent volume (1 mL of methylene blue vs. 3 mL of the other agents), contact time, activation method, and chemical mechanism also differed; these differences are integral to the tested protocols and prevent isolation of the effects of the agents themselves. Residual dentin thickness was not measured. SEM and push-out outcomes were obtained from different teeth, precluding specimen-level correlation. Antimicrobial efficacy, collagen integrity, wettability, resin-tag formation, degree of conversion, residual nanoparticles, and nanoparticle biocompatibility were not assessed, and the magnetic properties and crystalline phase of the nanoparticles were not independently characterized. Because iron oxide nanoparticles are dark, particles retained on the canal walls, within dentinal tubules, or in lateral canals after the final rinse could discolor the tooth over time, a concern analogous to that associated with silver-containing root canal materials; particle clearance and tooth color stability were not assessed and should be evaluated before clinical use. Likewise, cytotoxicity, nanoparticle migration, and periapical tissue responses were not evaluated, so no conclusion on clinical safety can be drawn; material composition and exposure conditions largely determine the biological response to endodontic materials [47]. Finally, SEM scoring was based on selected fields and an ordinal index; counting open and occluded dentinal tubules on the micrographs would provide more objective data. Although all SEM micrographs were acquired at ×10,000, the accelerating voltage varied (10–30 kV), which may have influenced the appearance of tubule orifices and the smear layer. Smear-layer scores were analyzed at the tooth level with nonparametric tests; an ordinal mixed-effects model of field-level scores, accounting for the clustering of fields within root halves and teeth, may provide additional information in future studies. No formal equivalence testing was performed. Future studies should also test the antibacterial efficacy of these protocols against endodontic pathogens such as Enterococcus faecalis, including in multispecies biofilm models. Future in vivo and ex vivo investigations are warranted to validate these findings under physiologically representative conditions.

## 5. Conclusions

Within the limitations of this in vitro investigation, we found no statistically significant difference in smear layer scores or push-out bond strength between NaOCl + SPIONs and the NaOCl + EDTA control at the cervical and middle thirds, whereas NaOCl + SPIONs yielded lower smear layer scores and higher push-out bond strength at the apical third, with no significant regional differences. NaOCl + SPIONs may therefore be regarded as a promising experimental final post-space treatment that requires further validation. Nevertheless, further well-designed in vitro and in vivo studies are warranted to substantiate these findings before clinical translation.

## Figures and Tables

**Table 1:** SL removal evaluation from post-space walls following different conditioning protocols (n = 10 teeth per group).

Final Conditioning Protocols	Median (IQR) Cervical	Median (IQR) Middle	Median (IQR) Apical	Kruskal–Wallis H(3) (C/M/A)
Group 1: EDTA	1.30 (1.06–1.35) ^a,A^	1.35 (1.03–1.55) ^a,A^	2.40 (2.36–2.77) ^b,B^	32.1/29.3/29.9 (all *p* < 0.001)
Group 2: aPDT-MB	2.60 (2.41–2.83) ^c,A^	2.72 (2.51–2.91) ^c,A^	3.54 (3.30–3.88) ^d,B^	
Group 3: SPIONs	1.20 (1.08–1.31) ^a,A^	1.25 (1.13–1.49) ^a,A^	1.32 (1.10–1.65) ^a,A^	
Group 4: ACV	1.93 (1.80–2.07) ^b,A^	2.10 (1.72–2.28) ^b,A^	3.06 (2.81–3.25) ^c,B^	

ACV = apple cider vinegar; aPDT-MB = antimicrobial photodynamic therapy with methylene blue; SPIONs = superparamagnetic iron oxide nanoparticles; EDTA = ethylenediaminetetraacetic acid; IQR = interquartile range. Different lowercase superscripts (a, b, c, d) within the same column indicate statistically significant intergroup differences (Kruskal–Wallis followed by Dunn’s test with Bonferroni adjustment; *p* < 0.05). Different uppercase superscripts (A, B) within the same row indicate statistically significant differences across root thirds (Friedman test followed by Wilcoxon signed-rank tests with Bonferroni adjustment; *p* < 0.05). Values are medians (25th–75th percentiles) of ten tooth-level scores per group; each tooth-level score is the mean of six field scores (three fields × two root halves), averaged across two examiners (12 integer ratings per root third), on the 1–5 Hülsmann scale. The percentiles describe the spread of the tooth-level scores and were not averaged further. Medians and percentiles are interpolated (grouped) values computed in SPSS. C = cervical; M = middle; A = apical. Exact test statistics: Kruskal–Wallis, cervical H(3) = 32.1, middle H(3) = 29.3, apical H(3) = 29.9 (all *p* < 0.001); Friedman, Group 1 χ^2^(2) = 15.80, Group 2 χ^2^(2) = 20.00, Group 4 χ^2^(2) = 18.20 (all *p* < 0.001), Group 3 χ^2^(2) = 3.80 (*p* = 0.150). n = 10 independent teeth per group; the tooth is the experimental unit. Q1, Q3 and effect sizes are given in Supplementary Tables S2 and S3.

**Table 2:** PBS (MPa) of GFP following different final post-space treatment protocols (n = 10 teeth per group).

Groups	Final Conditioning Protocols	Cervical Third Mean ± SD (MPa)	Middle Third Mean ± SD (MPa)	Apical Third Mean ± SD (MPa)
Group 1	NaOCl + EDTA	10.90 ± 0.81 ^a,A^	10.75 ± 0.77 ^a,A^	7.20 ± 0.61 ^b,B^
Group 2	NaOCl + aPDT-MB	6.10 ± 0.58 ^c,A^	5.80 ± 0.63 ^c,A^	3.90 ± 0.52 ^d,B^
Group 3	NaOCl + SPIONs	11.20 ± 0.84 ^a,A^	10.95 ± 0.79 ^a,A^	10.70 ± 0.76 ^a,A^
Group 4	NaOCl + ACV	7.90 ± 0.65 ^b,A^	7.60 ± 0.71 ^b,A^	6.10 ± 0.58 ^c,B^

ACV = apple cider vinegar; aPDT-MB = antimicrobial photodynamic therapy with methylene blue; SPIONs = superparamagnetic iron oxide nanoparticles; EDTA = ethylenediaminetetraacetic acid; MPa = megapascals. PBS was calculated as the load at failure divided by the bonded cement–dentin area (Equations (1) and (2)). Different lowercase superscript letters within the same column indicate statistically significant differences between groups (one-way ANOVA with Tukey’s HSD test at each root level; *p* < 0.05). Different uppercase superscript letters within the same row indicate statistically significant differences across root thirds (Bonferroni-adjusted paired comparisons; *p* < 0.05). Two-way mixed ANOVA: protocol, F(3, 36) = 176.4, ηp^2^ = 0.94; root level, F(1.5, 53.2) = 276.0, ηp^2^ = 0.89; protocol × root level, F(4.4, 53.2) = 33.1, ηp^2^ = 0.73; all *p* < 0.001 (within-subject effects Greenhouse–Geisser corrected, ε = 0.739; a single Mauchly’s test applies to both the root-level main effect and the interaction: W = 0.65, χ^2^(2) = 15.08, *p* < 0.001; Box’s M = 15.75, F(18, 4579.7) = 0.74, *p* = 0.77). Each value is the mean ± SD of ten teeth (one slice per tooth per root third, i.e., 10 teeth and 10 slices per cell); SDs therefore reflect between-tooth variation. Superscripts were verified against Tukey’s HSD comparisons at each root level and Bonferroni-adjusted paired comparisons within each protocol. SPIONs vs. EDTA (Tukey-adjusted): cervical *p* = 0.794, middle *p* = 0.927, apical *p* < 0.001. All pairwise comparisons, with exact adjusted *p*-values and 95% CIs, are given in Supplementary Table S1.

**Table 3:** Failure-mode distribution (counts) after push-out testing, by final post-space treatment protocol and root third.

Protocol	Root Third	AD-PC	AD-CD	CO-C	CO-D	CO-P	Admixed	Total
Group 1: EDTA	Cervical	0	1	2	3	1	3	10
	Middle	1	0	1	4	1	3	10
	Apical	1	3	1	2	0	3	10
	All thirds	2	4	4	9	2	9	30
Group 2: aPDT-MB	Cervical	1	3	1	2	0	3	10
	Middle	1	4	0	2	0	3	10
	Apical	1	5	0	1	0	3	10
	All thirds	3	12	1	5	0	9	30
Group 3: SPIONs	Cervical	0	1	2	4	1	2	10
	Middle	1	1	2	3	1	2	10
	Apical	1	2	1	4	0	2	10
	All thirds	2	4	5	11	2	6	30
Group 4: ACV	Cervical	0	1	1	3	0	5	10
	Middle	1	1	1	2	0	5	10
	Apical	1	2	1	1	0	5	10
	All thirds	2	4	3	6	0	15	30

AD-PC = adhesive failure at the post–cement interface; AD-CD = adhesive failure at the cement–dentin interface; CO-C, CO-D, CO-P = cohesive failure within cement, dentin, or post, respectively; Admixed = adhesive and cohesive features on the same slice. Counts are per slice (10 teeth per group; one slice per root third). No specimens were debonded before push-out testing.

## Data Availability

The data are available upon request to the authors.

## Supplementary Materials

The following supporting information can be downloaded at: https://www.mdpi.com/article/10.3390/polym18212762/s1. Supplementary Table S1. Pairwise comparisons of push-out bond strength between final post-space treatment protocols at each root third (Tukey’s HSD; n = 10 teeth per group). Supplementary Table S2. Descriptive statistics for smear layer scores by group and root third (n = 10 teeth per group). Supplementary Table S3. Omnibus test statistics and effect sizes for smear layer scores.
