# Supplementary material for: Effects of Photodynamic, Nanoparticle-Based, and Chemical Final Post-Space Conditioning on Smear Layer Removal and Push-Out Bond Strength of Glass Fiber-Reinforced Composite Posts
Source: Polymers (Basel). 2026 Nov 6;18(21):2762. doi: 10.3390/polym18212762 (PMC13766121; doi:10.3390/polym18212762)
Supplement: Supplementary file 1 [file polymers-18-02762-s001.zip › polymers-4623892-supplementary.pdf]

Supplementary Table S1. Pairwise comparisons of push-out bond strength between final post-space treatment protocols at each root third (Tukey's HSD; n = 10 teeth per group).

Root third	Comparison	Mean difference (MPa)	95% CI (MPa)	Adjusted p
Cervical	EDTA vs. aPDT-MB	4.80	3.92 to 5.68	< 0.001
	EDTA vs. SPIONs	−0.30	−1.18 to 0.58	0.794
	EDTA vs. ACV	3.00	2.12 to 3.88	< 0.001
	aPDT-MB vs. SPIONs	−5.10	−5.98 to −4.22	< 0.001
	aPDT-MB vs. ACV	−1.80	−2.68 to −0.92	< 0.001
	SPIONs vs. ACV	3.30	2.42 to 4.18	< 0.001
Middle	EDTA vs. aPDT-MB	4.95	4.07 to 5.83	< 0.001
	EDTA vs. SPIONs	−0.20	−1.08 to 0.68	0.927
	EDTA vs. ACV	3.15	2.27 to 4.03	< 0.001
	aPDT-MB vs. SPIONs	−5.15	−6.03 to −4.27	< 0.001
	aPDT-MB vs. ACV	−1.80	−2.68 to −0.92	< 0.001
	SPIONs vs. ACV	3.35	2.47 to 4.23	< 0.001
Apical	EDTA vs. aPDT-MB	3.30	2.55 to 4.05	< 0.001
	EDTA vs. SPIONs	−3.50	−4.25 to −2.75	< 0.001
	EDTA vs. ACV	1.10	0.35 to 1.85	0.002
	aPDT-MB vs. SPIONs	−6.80	−7.55 to −6.05	< 0.001
	aPDT-MB vs. ACV	−2.20	−2.95 to −1.45	< 0.001
	SPIONs vs. ACV	4.60	3.85 to 5.35	< 0.001

Mean difference = first protocol minus second protocol. CIs and p values are Tukey-adjusted for the six comparisons at each root third (one-way ANOVA; error df = 36). Homogeneity of variances was confirmed at each root third (Levene's test, $p \geq 0.36$; Bartlett's test: cervical $\chi^2(3) = 1.57$, $p = 0.666$; middle $\chi^2(3) = 0.52$, $p = 0.915$; apical $\chi^2(3) = 1.37$, $p = 0.712$). EDTA = 17% ethylenediaminetetraacetic acid; aPDT-MB = methylene blue-mediated antimicrobial photodynamic therapy; SPIONs = superparamagnetic iron oxide nanoparticles; ACV = apple cider vinegar.

Supplementary Table S2. Descriptive statistics for smear layer scores by group and root third (n = 10 teeth per group).

Group	Root third	Median	Q1	Q3	IQR
G1: EDTA	Cervical	1.30	1.06	1.35	0.29
	Middle	1.35	1.03	1.55	0.52
	Apical	2.40	2.36	2.77	0.41
G2: aPDT-MB	Cervical	2.60	2.41	2.83	0.42
	Middle	2.72	2.51	2.91	0.40
	Apical	3.54	3.30	3.88	0.58
G3: SPIONs	Cervical	1.20	1.08	1.31	0.23
	Middle	1.25	1.13	1.49	0.36
	Apical	1.32	1.10	1.65	0.55
G4: ACV	Cervical	1.93	1.80	2.07	0.27
	Middle	2.10	1.72	2.28	0.56
	Apical	3.06	2.81	3.25	0.44

Q1 = 25th percentile; Q3 = 75th percentile; IQR = interquartile range (Q3 − Q1). Scores on the 1–5 Hülsmann scale; tooth-level values as defined in Table 1. Percentiles are interpolated (grouped) values computed in SPSS. EDTA = ethylenediaminetetraacetic acid; aPDT-MB = antimicrobial photodynamic therapy with methylene blue; SPIONs = superparamagnetic iron oxide nanoparticles; ACV = apple cider vinegar.

Supplementary Table S3. Omnibus test statistics and effect sizes for smear layer scores.

Comparison	Test	Statistic	df	p	Effect size
Intergroup, cervical	Kruskal–Wallis	H = 32.1	3	< 0.001	$\varepsilon^2 = 0.82$
Intergroup, middle	Kruskal–Wallis	H = 29.3	3	< 0.001	$\varepsilon^2 = 0.75$
Intergroup, apical	Kruskal–Wallis	H = 29.9	3	< 0.001	$\varepsilon^2 = 0.77$
Intra-group, G1: EDTA	Friedman	$\chi^2 = 15.80$	2	< 0.001	W = 0.79
Intra-group, G2: aPDT-MB	Friedman	$\chi^2 = 20.00$	2	< 0.001	W = 1.00
Intra-group, G3: SPIONs	Friedman	$\chi^2 = 3.80$	2	0.150	W = 0.19
Intra-group, G4: ACV	Friedman	$\chi^2 = 18.20$	2	< 0.001	W = 0.91

ε^2 = epsilon-squared, H/(N − 1), N = 40; W = Kendall's coefficient of concordance, $\chi^2/[n(k-1)]$, n = 10, k = 3. Post hoc comparisons: Dunn's test (intergroup) and Wilcoxon signed-rank tests (intra-group), both with Bonferroni adjustment (p < 0.05). EDTA = ethylenediaminetetraacetic acid; aPDT-MB = antimicrobial photodynamic therapy with methylene blue; SPIONs = superparamagnetic iron oxide nanoparticles; ACV = apple cider vinegar.
